# *De novo LAMP2* insertion mutation causes cardiac-only Danon disease: A case report

**DOI:** 10.3389/fcvm.2022.899283

**Published:** 2022-09-16

**Authors:** James Jiqi Wang, Bo Yu, Xiuli Song, Hong Wang

**Affiliations:** ^1^Division of Cardiology, Department of Internal Medicine, Tongji Medical College, Tongji Hospital, Huazhong University of Science and Technology, Wuhan, China; ^2^Hubei Key Laboratory of Genetics and Molecular Mechanism of Cardiological Disorders, Tongji Medical College, Tongji Hospital, Huazhong University of Science and Technology, Wuhan, China; ^3^Genetic Diagnostics Center, Tongji Medical College, Tongji Hospital, Huazhong University of Science and Technology, Wuhan, China

**Keywords:** Danon disease, Sanger sequencing, genetic diagnosis, *LAMP2*, hypertrophy

## Abstract

Danon disease is a rare disease caused by glycogen storage lysosomal disorder. It is related to the pathogenic mutation of the *LAMP2* gene. In this case report, we present a patient with a novel pathogenic mutation (c.764_765insGA) with cardiac-only symptoms. Her family members do not carry the same mutation she does, suggesting this is a *de novo* mutation. Further tests revealed vacuoles and glycogen disposition in the patient's heart tissue and a significant decrease in LAMP2 protein expression. Protein structure remodeling of LAMP2 predicted that the mutant protein has conformational change lacking an important transmembrane domain, subsequently causing protein destabilization.

## Introduction

Danon disease (OMIM: 300257) is an X-linked dominant disorder caused by a defect of the lysosome-associated membrane protein 2 (LAMP2) gene (1). LAMP2 is essential in the progress of the autophagosome maturation (2). Mutations of *LAMP2* identified in Danon disease lead to splicing defects or protein truncation, which underlies LAPM2 deficiency in skeletal and cardiac muscles (1). Defect of *LAMP2* caused impaired autophagosome-lysosome fusion and degradation, which leads to failure of cellular autophagy and accumulation of glycogen granules and autophagic vacuoles (3).

Danon disease is clinically characterized by a triad of skeletal myopathy, intellectual disability, and cardiomyopathy (4). Due to haploinsufficiency, male patients are usually more severely affected than female patients and are often the proband of this disease (5). The impact on the skeletal muscle manifests as myalgia, poor exercise tolerance, and fatigue (1). Serum creatine kinase (CK) levels are often elevated in male patients with skeletal muscle involvement and in female patients, serum CK levels are usually normal or mildly elevated (1). Mild to moderate intellectual disabilities are found in 70%-100% of male patients and 50% of female patients exhibit mild mental disorder (6, 7). Patients with Danon disease mainly manifest conduction abnormalities and cardiomyopathy. Wolf-Parkinson-White syndrome predominates in electrocardiograph (ECG) abnormalities (69% of patients) and others include supraventricular tachycardia, delta waves, high precordial voltage, and complete ventricular block (AVB) (8). Cardiomyopathy is the predominant and most life-threatening manifestation for a patient with Danon disease, including dilated cardiomyopathy and hypertrophic cardiomyopathy (HCM). Cardiac syndromes include chest tightness, heart murmur, fatigue, or palpation. In male patients, cardiac symptoms usually began in infancy/childhood or adolescence, while females present with slower progression and later onset of the disease. In some female patients, the cardiac disease may act as an isolated clinical feature (73%) (2).

The exact prevalence of Danon disease is not clear. The diagnosis of Danon disease is gradually increasing with the development of gene testing. Unfortunately, cardiac transplantation and implantable cardioverter defibrillator (ICD) are the only therapeutic interventions for Danon disease (9, 10). According to a study, without heart transplantation, the mean age of death in males is 19.0 ± 8.0 and 34.6 ± 15.5 in females (6). Thus, an early diagnosis and clinical intervention are essential to prevent the lethal prognosis of this disease.

Herein we report a Danon disease case carrying a novel frameshift (c.764_765insGA) *LAMP2* variant who only manifested cardiac symptoms. Her family members did not share her mutation or phenotype, suggesting that this was a *de novo* mutation.

## Materials and methods

### Whole exome sequencing

The proband and her family members' genome DNA were extracted from their peripheral blood *via* QIAamp DNA Mini Kit (Qiagen, Germany) and quantified with the Nanodrop 2000 (Life Technology, USA).

To thoroughly identify the potentially pathogenic gene, whole exome sequencing was carried out using the proband's DNA. Qualified samples were exome sequenced using the SureSelect Human All Exon library kit (Agilent, USA) and Novaseq 6000 or Hiseq Xten platform in the paired-end mode of 150 base pairs (Illumina, California, USA). The whole exome sequencing (WES) data were processed in accordance with the best practice of The Genome Analysis Toolkit. The resulting variants were annotated using ANNVAR. Rare variants were defined as those with a minor allele frequency of <0.001 in East Asian databases from ExAC, The 1000 Genomes Project, and gnomAD.

### Sanger sequencing

Potentially identified mutation and low coverage regions (<20 folds) were verified *via* Sanger sequencing with specific primers. Validated mutations were also Sanger sequenced in the patient's relatives and extra 800 unrelated Chinese health controls.

### Pathological analysis and immunohistochemistry analysis

Biopsy specimens were obtained from the patient's left ventricle. Hematoxylin-eosin (HE) and Periodic acid-Schiff (PAS) staining were performed to investigate the patient's myocardial structure and glycogen disposition. For immunohistochemistry, a primary antibody against LAMP2 (1:100 dilution) was obtained from Abclonal (China, A14017) and a secondary antibody (goat anti-rabbit, 1:2000 dilution) was obtained from Abcam (England, ab205718).

### Proteome expression analysis

We performed proteomic analysis to further investigate protein expression caused by this mutation using white blood cells from the patient's peripheral blood. Workflow of blood samples prepared before the proteomic measurement is described elsewhere (11). Samples were measured using an LC-MS instrument consisting of an EASY-nLC 1200 ultra-high-pressure system (Thermo Fisher Scientific) coupled *via* a nano-electrospray ion source to Fusion Lumos Orbitrap (Thermo Fisher Scientific). The mass spectrometry proteomics data have been deposited to the ProteomeXchange Consortium (http://proteomecentral.proteomexchange.org) *via* the iProX partner repository with the dataset identifier PXD034265. LAMP2/ACTB ratio was used to compare the expression of LAMP2 between the patient and healthy controls.

### Protein structure remodeling

We predicted the structure of the mutant protein using a deep learning technique. The specific protein structural modeling open-source code using AlphaFold Protein Structure Database is available at (https://github.com/deepmind/alphafold) (DeepMind Technologies, USA). Figures were presented *via* UCSF ChimeraX (RBVI, USE) (12).

## Results

### Case report

**Figure 1:**
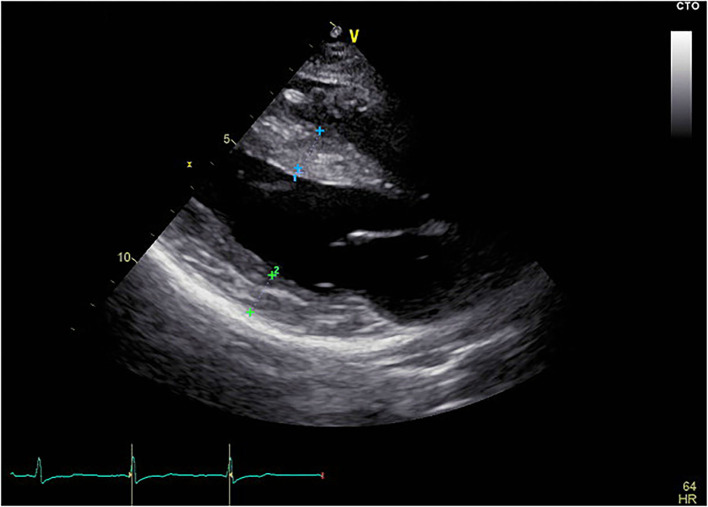
Echocardiogram of the patient. The echocardiogram showed thickened interventricular septum and left ventricular posterior wall. Blue cross marked part: interventricular septum, thickness was 1.4 cm; Green cross marked part: posterior wall of left ventricle, thickness was 1.4 cm; Yellow scale bar shows length in cm.

**Figure 2:**
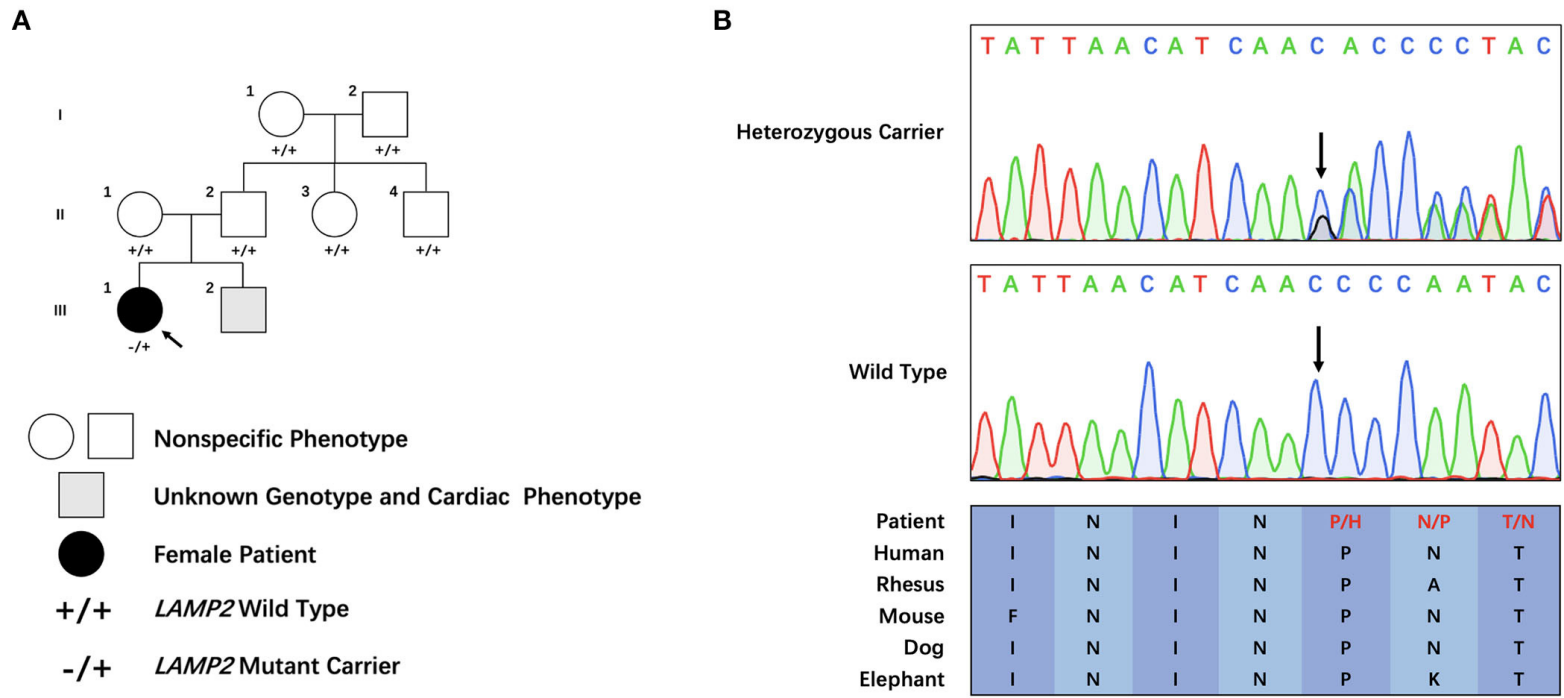
Clinical and genetic characteristics of the pedigree. **(A)** Family tree of the cardiac-only Danon disease pedigree. **(B)** Sanger sequencing of the family. Colored blocks indicate evolutionary conservation of the cluster across multiple species.

The proband was a 25-year-old female who complained of intermittent chest distress and shortness of breath for half a month before being admitted to our hospital. Symptoms were relieved automatically after a few minutes. According to the patient, she had experienced similar cardiac symptoms in the past few years with no significant inducement. The patient had mild myopia which can be corrected with glasses. Intelligence Quotient (IQ) test showed normal results (IQ: 111). She denied having symptoms of skeletal muscle and the electromyogram did not show abnormal electrical activity. Her parents (father, 52 y, and mother, 50 y) did not show any cardiac, skeletal muscle, or mental disorder and neither did her 6-year-old brother. On admission, her body temperature was 36.3°C, pulse 60 bpm, respiratory rate 20/min, and blood pressure 90/61 mmHg. Blood biochemistry tests indicated slightly elevated aspartate aminotransferase (AST, 42 U/L, normal range: 0–32 U/L), alanine aminotransferase (ALT, 38 U/L, normal range: 0–33 U/L), and normal level of creatine kinase (CK, 75 U/L, normal range: 0–170 U/L). Blood routine examination revealed elevated white blood cells (WBC, 16.35^*^10∧9/L) and neutrophils (12.45^*^10∧9/L). Myocardial markers showed significantly elevated amino terminal brain natriuretic peptide precursor (NT-proBNP, 2,276 pg/ml) and hypersensitive cardiac troponin I (hscTNI, 3,171.7 pg/ml). Respiratory pathogen antibody test showed positive influenza A virus IgM, mycoplasma pneumonia IgG, and chlamydia IgG. The patient was first diagnosed with acute myocarditis and was treated with mechanical circulatory support measurement (intra-aortic balloon counterpulsation) in accordance with the procedures previously reported because of shock (13). The patient's vital signs stabilized after 8 days. However, during hospitalization, her echocardiogram (Figure 1) indicated a thickness of the interventricular septum (basal segment: 9 mm; middle segment: 14 mm; apical segment: 14 mm), left ventricular posterior wall (14 mm), and apical segment of the anterolateral wall (11 mm). Holter monitor and ECG (Supplementary Figure 1) showed a short PR interval (76 ms), “delta wave,” and lowered ST segment (V4–V6), suggesting preexcitation of the ventricle. In order to differentiate from myocardial edema which also presents a transient thickness of cardiac muscle, another echocardiogram was performed before discharge and the result still showed a thickened interventricular septum (basal segment: 9 mm; middle segment: 16 mm) and left ventricular posterior wall (11 mm). For genetic testing, genomics was extracted from the patient's peripheral blood and genetic sequencing revealed a novel frameshift heterozygous mutation (c.764_765insGA) of *LAMP2*. However, her mother, father, uncle, and aunt did not have the same mutation (Figure 2A). Her 6-year-old younger brother had normal development and did not experience chest distress, shortness of breath, palpitation, or exhibit other cardiac symptoms. The patient's family refused echocardiogram and genetic testing for the patient's brother (Figure 2A).

### Genetic analysis

Through filtering and Sanger sequencing, we found a pathogenic heterozygous frameshift mutation, c.764_765insGA, following the ACMG guideline (14). We did not identify any other potentially pathogenic variant of CNVs of known ion channelopathy or hypertrophic cardiomyopathy-related genes. Neither was it found in the extra 800 unrelated Chinese healthy controls. Moreover, this mutation was absent in public databases (Clinvar: https://www.ncbi.nlm.nih.gov/clinvar/, ExAC: http://exac.broadinstitute.org/ and HGMD: http://www.hgmd.cf.ac.uk/ac/search.php) is evolutionary, and highly conserved across multiple species (Figure 2B). The mutation was not identified in her parents or other family members, which suggested this novel frameshift mutation was a *de novo* one.

### Pathological and immunohistochemical analysis

**Figure 3:**
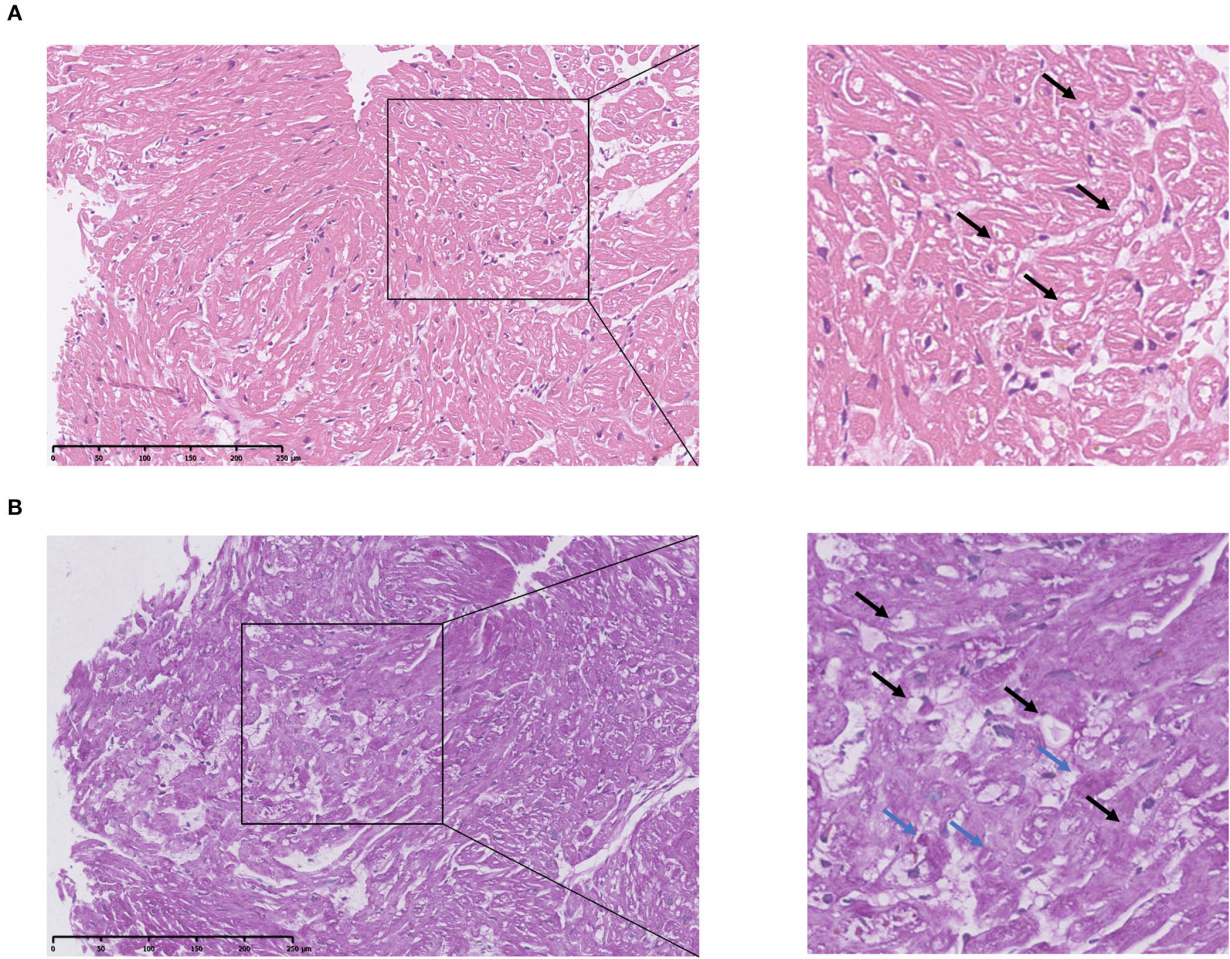
Pathological analysis of patient's cardiac muscle specimen. **(A)** HE staining of patient's heart tissue. The square shows destructed cardiac cell structure. Arrows indicate small vacuoles within cells. **(B)** PAS staining of patient's heart tissue. Blue arrows indicate glycogen disposition within the muscle fiber. Black arrows indicate small vacuoles within cells.

**Figure 4:**
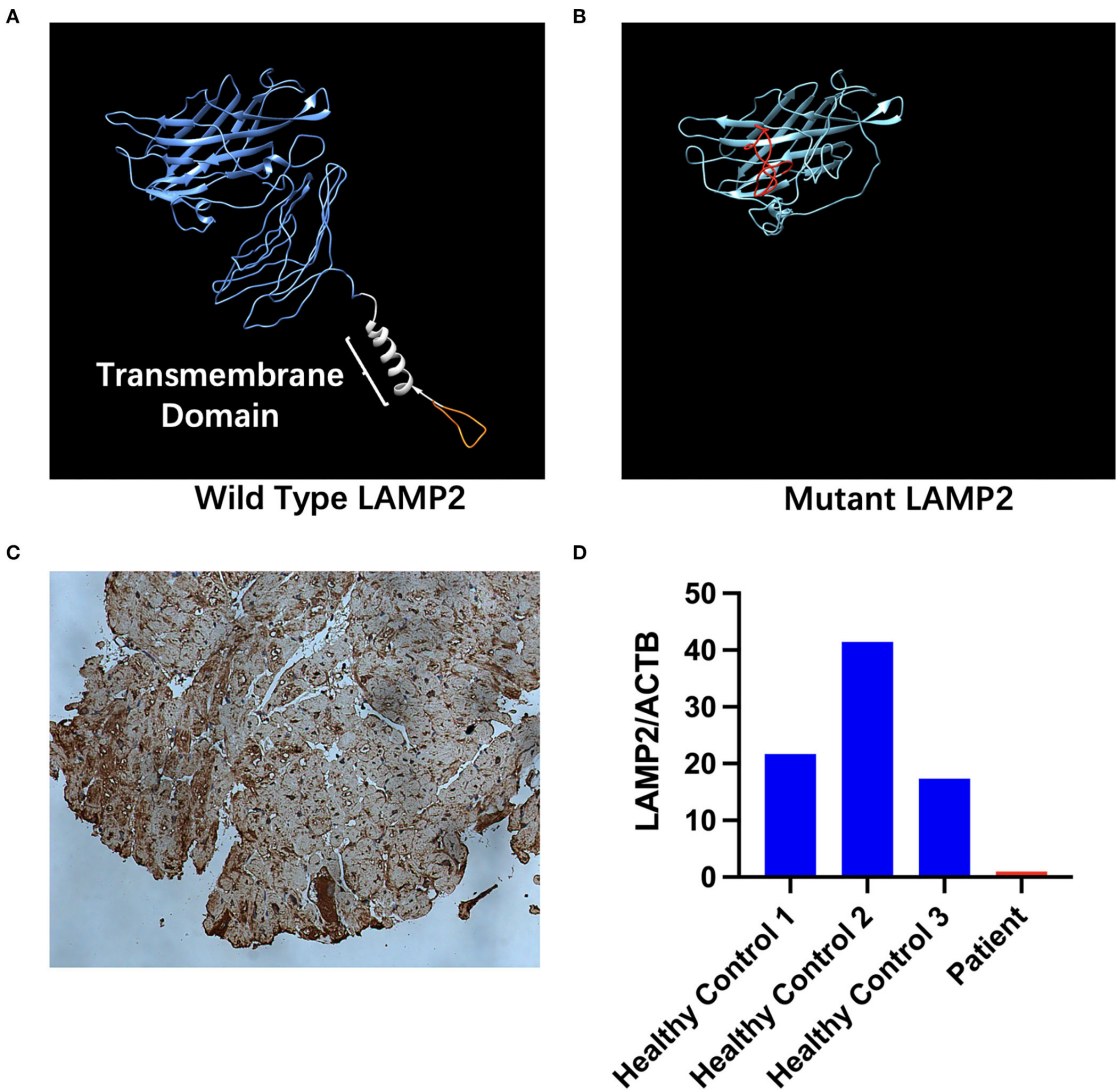
Structure and expression changes caused by mutant *LAMP2*. **(A)** *in-silico* structural modeling of wild-type LAMP2. The orange part shows the part that is truncated in the mutant LAMP2. The blue segment indicates the lumenal part of LAMP2. The white segment indicates the transmembrane part of LAMP2. The orange segment indicates the cytoplasmic part of LAMP2. **(B)** *In-silico* structural modeling of mutant LAMP2. The red segment indicates the mistranslated segment caused by the insertion mutation. The transmembrane helical domain is missing. **(C)** Immunohistochemical analyses for LAMP2. **(D)** Relative expression of LAMP2 in patient's and healthy controls' peripheral blood measured *via* proteomic analysis.

HE staining of the endocardial biopsy sample section revealed ambiguous cell boundary, and karyopyknosis of the cell nucleus (Figures 3A,B), indicating destruction of myocardiocytes. The PAS staining showed abnormal cell structure, small vacuoles, and glycogen disposition within muscle fibers (Figures 3A,B), which is consistent with the canonical histological feature of Danon disease. Immunohistochemical (IHC) staining of the patient's myocardial slice against LAMP2 showed high and low expressing patches (Figure 4C).

### Proteome expression analysis

Proteomic analysis revealed a decreased expression of the patient's LAMP2 compared with other healthy controls. LAMP2 was normalized by internal reference (ACTB). LAMP2 Expression of the patient was significantly lower than healthy controls (Figure 4D, Supplementary Table 1). On average, healthy controls express 26.83 ± 12.87 times more LAMP2 than patients carrying c.764_765insGA mutation.

### Protein structure remodeling

The c.764_765insGA inserting mutation causes a mistranslation in the downstream and premature termination to 281-amino-acid residues. Significant differences were found between the mutant and wild-type LAMP2 (Figures 4A,B); i.e., the truncated mutant protein lacks transmembrane helical domain and cytoplasmic domain, leading to abnormal protein function.

## Discussion

To date, more than 200 pathogenic mutations have been reported, covering exon 1 to exon 9. Through genetic sequencing, we identified a novel *LAMP2* frameshift (c.764_765insGA) mutation. The frameshift mutation in our patient caused premature termination of protein translation 46 amino acids forward. Unlike most other cases, the proband of this case is female. Her parents and relatives, however, who are all over 50 years old, did not share the same mutation or symptom with her, and neither did her 6-year-old brother. These findings suggest that this is a *de novo* pathogenic mutation. The mutation we revealed was absent in the unrelated 800 healthy controls, the Exome Aggregation Consortium (ExAc), ClinVar, and HGMD databases. According to the ACMG guideline and standard, this mutation should be categorized as a pathogenic mutation (PVS1+PS2+PM2). What is worth noting is that she was first presented with myocarditis, and we wondered if there was a potential correlation between Danon disease and myocarditis. However, we did not find any cases of Danon diseases combined with myocarditis. Thus, to the best of our knowledge, this is the first Danon disease case that presented first with myocarditis. Whether there is a connection between the susceptibility of myocarditis and the destruction of heart structure requires further investigation. What caught our attention is that the primary diagnosis of the patient was not Danon disease. If a gene diagnosis test was not taken into consideration, her pathogenic mutation may not have been revealed and may pass on to her offspring, which would cause a huge emotional and economic burden to this family. This reflects the important role genetic test plays in the diagnosis of patients.

As the main components of the lysosomal membrane LAMP2 are thought to protect the lysosomal membrane from hydrolytic enzymes, studies have proven a more crucial role of LAMP2 compared to LAMP1 (15). To further investigate the impact of this mutation, a heart biopsy was obtained from her left ventricle. HE staining of the section revealed unstructured cardiac cells (Figure 3A). PAS staining showed small vacuoles and glycogen disposition within cardiac muscle fibers (Figure 3B), which was similar to previously reported pathological features of the Danon disease (2). IHC staining of the heart tissue showed high and low expressing patches of LAMP2 (Figure 4C). Protein structure remodeling *via* deep learning technique predicted that the mutation lacks the vital cross membrane domain which may lead to an unstabilized protein structure. Further proteomic analysis revealed a considerable decrease in the patient's *LAMP2* expression (Figure 4D), which is in accordance with protein structure prediction, contributing to the deficiency of functioning LAMP2. Unlike most cases, the proband of this case is female. Her symptoms did not cover all the classical clinical triad mentioned above, but only presented with cardiac hypertrophy and preexcitation of the ventricle. This is probably due to the haploinsufficiency of her heterozygous mutation. This is in accordance with previously reported female Danon disease patients, where only one-third of female patients had skeletal myopathy and 50% had cognitive disorders (1). Haploinsufficiency is a very important finding regarding the pathophysiology of female Danon disease patients. Sugie et al. reported a female Danon disease patient whose LAMP2 was significantly decreased (only 1/6 compared to control) and had severe cardiomyopathy but no skeletal myopathy while her son had both heart and skeletal muscle involvement (16). In 2016 they reported another Danon disease female patient whose LAMP2 decreased by 50% (LAMP2-haploinsufficiency) (17). These female Danon disease patients with heterozygous pathogenic *LAMP2* mutation shared similar LAMP2 expression features and symptoms with the patient in this report.

To date, the ClinVar database included 29 frameshift mutations of the *LAMP2* gene. Compared with missense mutations, frameshift mutations generally cause more severe damage to the protein's length and structure. Thus, all the reported 29 frameshift mutations are categorized as “likely pathogenic” or “pathogenic.”

In conclusion, we identified a *de novo* frameshift mutation in a Danon disease female patient that only presented with cardiac hypertrophy and preexcitation. To the best of our knowledge, this mutation has not been reported earlier. This finding enriches the pathogenic gene spectrum of *LAMP2* and facilitates future genetic counseling and genetic diagnosis.

## Data availability statement

The datasets presented in this study can be found in online repositories. Proteomic data presented in this study can be found *via* the link at: http://proteomecentral.proteomexchange.org/cgi/GetDataset?ID=PXD034265. Exome sequencing in this study can be found *via* the following link: https://ngdc.cncb.ac.cn/gsa-human/browse/HRA002525.

## Ethics statement

The studies involving human participants were reviewed and approved by Ethics Committee of Tongji Hospital, Tongji Medical College, Huazhong University of Science and Technology. The patients/participants provided their written informed consent to participate in this study. Written informed consent was obtained from the individual(s) for the publication of any potentially identifiable images or data included in this article.

## Author contributions

HW designed and conceptualized this study. JW wrote this manuscript and conducted the pathological analysis of the patient's left ventricle tissue. BY conducted DNA isolation, whole exome sequencing, and revised the manuscript. XS participated in the clinical sample and data collection. All authors read and approved this manuscript.

## Funding

This study was supported by the National Natural Science Foundation of China (82100526).

## Conflict of interest

The authors declare that the research was conducted in the absence of any commercial or financial relationships that could be construed as a potential conflict of interest.

## Publisher's note

All claims expressed in this article are solely those of the authors and do not necessarily represent those of their affiliated organizations, or those of the publisher, the editors and the reviewers. Any product that may be evaluated in this article, or claim that may be made by its manufacturer, is not guaranteed or endorsed by the publisher.
