# Supplementary figures and images for: De novo LAMP2 insertion mutation causes cardiac-only Danon disease: A case report
Source: Front Cardiovasc Med. 2022 Sep 16;9:899283. doi: 10.3389/fcvm.2022.899283 (PMC9523138; doi:10.3389/fcvm.2022.899283)

**Supplementary Figure 1. Electrocardiogram of the patient.**

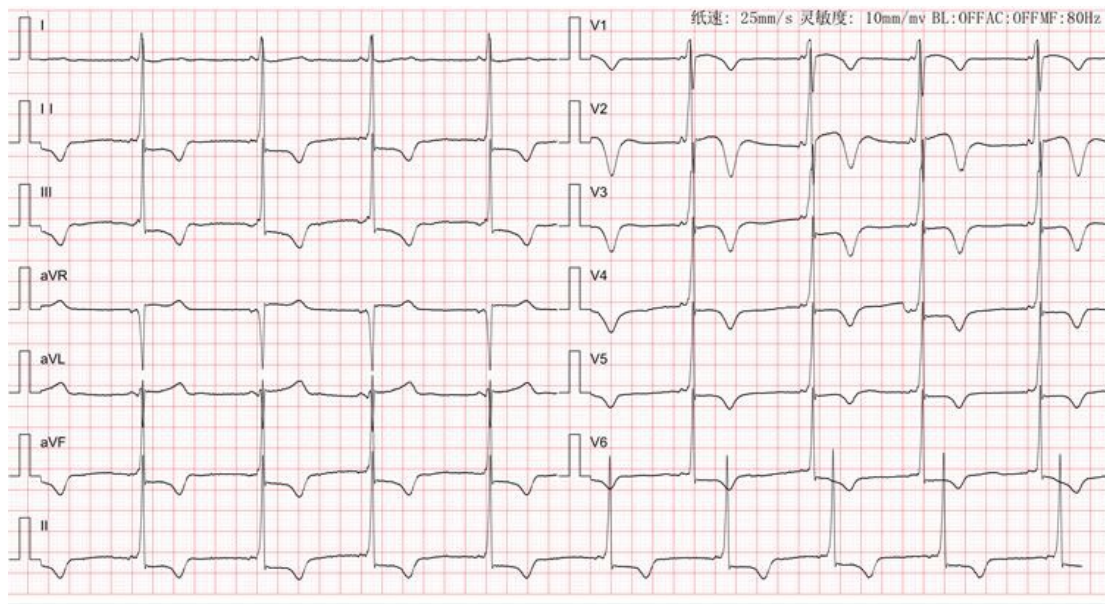

Supplement: Supplementary file 2 [file Image_1.pdf]
